# Bacteraemia with *Moryella indoligenes* and *Fastidiosipila sanguinis*: a case report

**DOI:** 10.1099/acmi.0.000108

**Published:** 2020-02-25

**Authors:** Sanne Kjær Hansen, Sandra Valborg Løfberg, Dorte Kassentoft Nielsen, Hanne Kobberø, Ulrik Stenz Justesen

**Affiliations:** ^1^​ Department of Clinical Microbiology, Odense University Hospital, Odense, Denmark; ^2^​ Internal Medicine and Emergency Department, Odense University Hospital, Svendborg, Denmark; ^3^​ Department of Urology, Odense University Hospital, Odense, Denmark

**Keywords:** *Moryella indoligenes*, *Fastidiosipila sanguinis*, bacteraemia

## Abstract

*
Moryella indoligenes* and *Fastidiosipila sanguinis* are obligate anaerobic Gram-positive bacteria that are rarely involved in human infections. We present the first case of bacteraemia with *M. indoligenes *, which was part of a co-infection with *F. sanguinis *. Both micro-organisms were identified by 16S rRNA gene sequencing and *M. indoligenes* was also identified by matrix-assisted laser desorption/ionization time-of-flight mass spectrometry (MALDI-TOF MS). Prostate cancer involving the bladder suggests that the urinary tract was the most likely primary site of infection.

## Introduction

Based on improved molecular diagnostics, including sequencing, the number of new species that have been associated with the human microbiome has been steadily increasing in recent years. However, for some of the species their natural habitat in the body is unknown and the pathogenesis of the infections has only been sparsely described. Consequently, we find it important to publish cases reporting infections with newly described species. In this paper we report the first case of bacteraemia with *Moryella indoligenes *, which was part of a co-infection with *Fastidiosipila sanguinis *.

## Case report

**Fig. 1.**
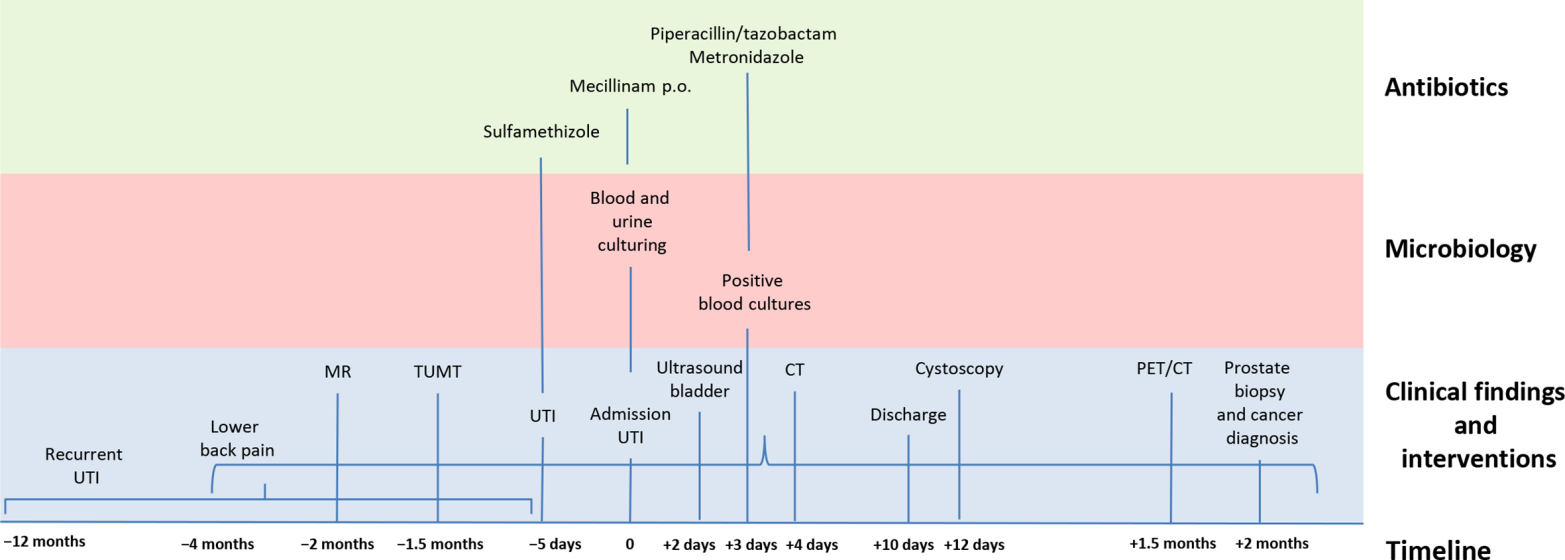
Case timeline. UTI, urinary tract infection; MR, magnetic resonance; TUMT, transurethral microwave therapy, CT, computed tomography; PET/CT, positron emission tomography with CT.

An older male was admitted to hospital with suspicion of a urinary tract infection. He had a medical history of prostatic hypertrophy complicated with recurrent urinary tract infections (Fig. 1). He also suffered from obstipation and progressing pain of the lower back, which had lasted for 4 months. A magnetic resonance scan of the lower back performed 2 months prior to admission was normal. He had also had transurethral microwave therapy performed and in the following period he used a urinary catheter or intermittent catheterization. Because of persistence of blood in the urine and suspicion of a urinary tract infection the patient was treated with sulfamethizole by his general practitioner. Despite treatment, the symptoms persisted and the patient was admitted to hospital for further examination.

On admission, the patient’s primary complaints were fever (38.9 °C) and persistent haematuria. The white blood cell count was 18.0×10^9^ l^−1^ and the C-reactive protein (CRP) was 11 mg l^−1^, rising to 72 mg l^−1^ over the next 2 days. The patient had an ultrasound scan of the bladder, which showed residual urine. A urinary catheter was inserted. Blood cultures, two anaerobic and two aerobic bottles (total of 40 ml, BacT/ALERT VIRTUO, bioMérieux SA, Marcy l´Étoile, France), and a urine sample was obtained before antibiotic treatment was changed from sulfamethizole to oral mecillinam (amdinocillin) 400 mg three times a day.

No pathogenic organisms were detected from the urine sample after culturing under aerobic conditions. After 3 days of incubation, growth was detected in the two anaerobic bottles. Gram-staining showed Gram-variable rods. Two days later, the growth of small transparent, smooth and convex colonies was detected on Brucella blood agar supplemented with hemin and vitamin K1 (Becton Dickinson GmbH, Heidelberg, Germany) grown under anaerobic conditions (80 % N_2_, 10 % CO_2_, 10 % H_2_). Bacterial identification was performed using matrix-assisted laser desorption/ionization time-of-flight mass spectrometry (MALDI-TOF MS; Microflex LT, BDAL revision no. 8 including 7854 MSPs, Bruker Daltonik GmbH, Bremen, Germany). *M. indoligenes* was identified from both blood culture bottles with a score of 2.08. The main spectrum profile used for bacterial identification of *M. indoligenes* was the library strain, ENR_0198ENR. This strain was incorporated in the library (Biotyper, Bruker) as part of the project ‘European Network of Rapid Identification of Anaerobes’ (ENRIA) [1].

**Fig. 2.**
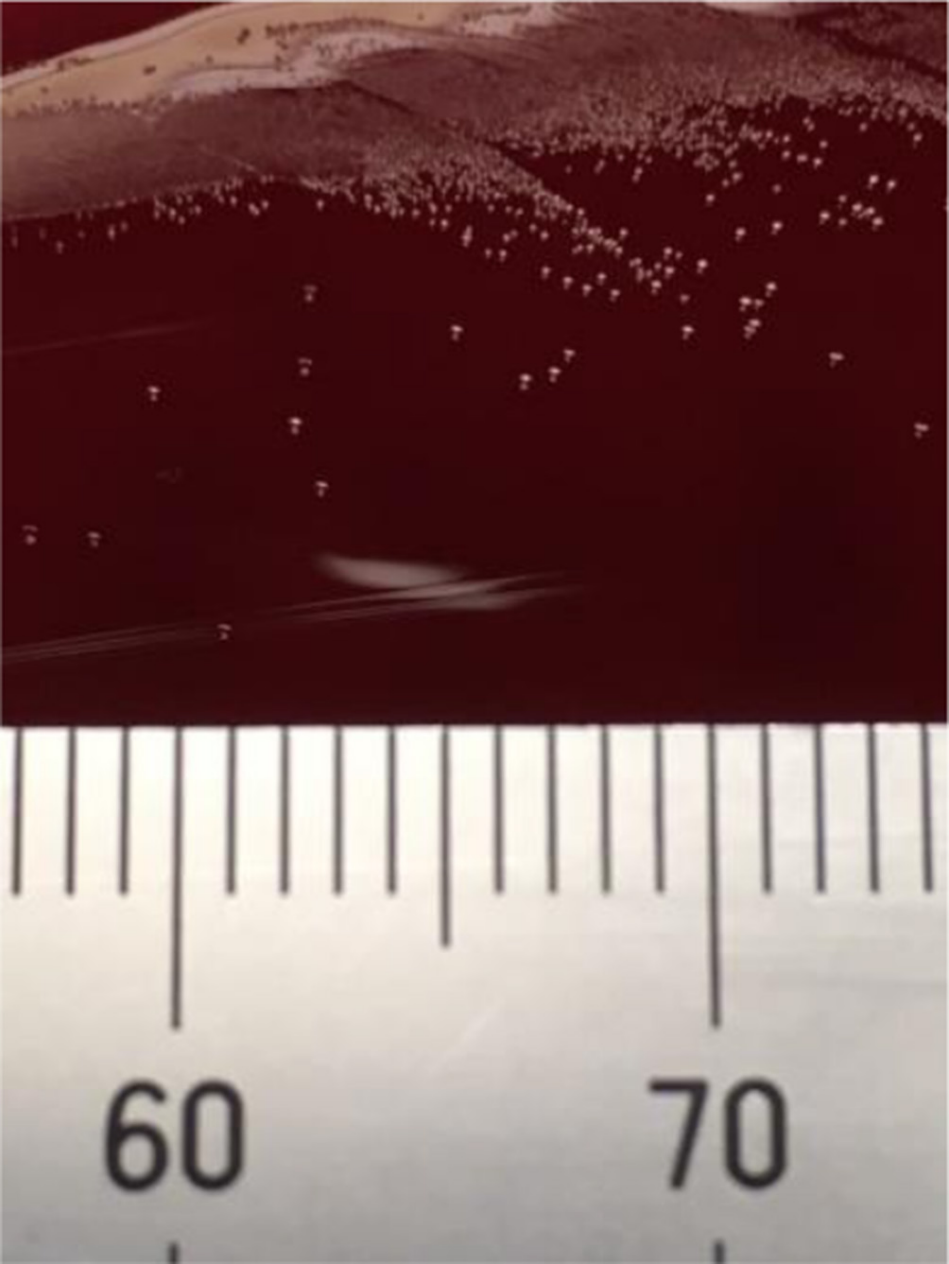
*
F. sanguinis* on Brucella blood agar.

**Fig. 3.**
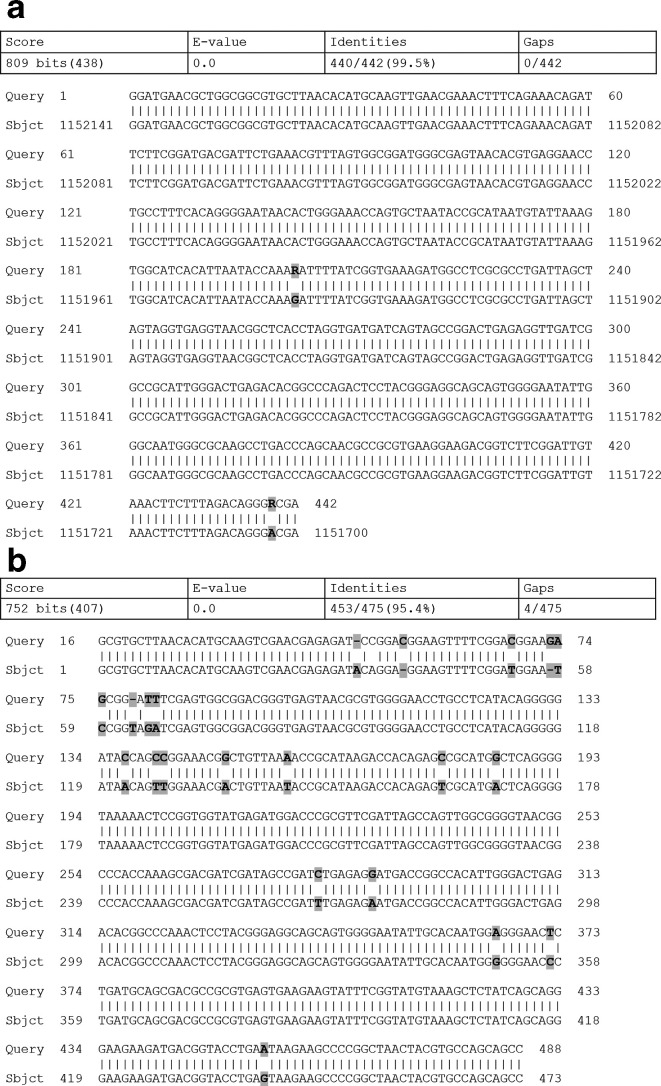
(a) Alignment of the partial 16S rRNA gene sequence from the case isolate of *F. sanguinis* with *F. sanguinis* strain CCUG 47711 chromosome, complete genome (sequence ID: CP027226). There are two mismatches at position 202 (G to R=A or G) and at position 439 (A to R) resulting in a match of 99.5 %. (b) Alignment of the sequence obtained from the mixed sequence from the case isolate using the Pathogenomix Ripseq software and database, of *M. indoligenes* with *M. indoligenes* strain MDA2477 (sequence ID: AF527773). There are several mismatches and gaps, resulting in a match of 95.4 %.

Antimicrobial susceptibility testing was primarily performed with an in-house disk diffusion method on Brucella blood agar and read after 24 and 48 h of incubation. Apparently, the isolate was resistant to metronidazole. However, a clear double zone was observed and the colonies close to the metronidazole disk were slightly smaller, a bit greyer and flatter than the colonies in the periphery (Fig. 2). Repeated identification of these colonies by MALDI-TOF resulted in a low score identification of several different species (score <1.7). Subcultures from the colonies close the metronidazole disk and from colonies in the periphery were performed on Brucella blood agar. Bacterial identification of both species using MALDI-TOF and 16S rRNA gene sequencing was performed. For both the isolates only 16S rRNA gene sequencing resulted in species identification. For the colonies growing close to the metronidazole disk *F. sanguinis* with a score of 99.5 was suggested (442 bp sequence, sequence ID: CP027226, coverage of 100%) using the Basic Local Alignment Search Tool (blast) (Fig. 3). For the colonies in the periphery, a mixed chromatogram was found, suggesting DNA from more than one species. Using the Pathogenomix Ripseq software and database the best hits were *F. sanguinis* with a score of 100, and a *Lachnospiraceae* species with a score of 99.5 [2]. The DNA sequence of the *Lachnospiraceae* species (sequence ID: NBBL01000005) was further investigated using blast. *M. indoligenes* was identified with a score of 95.4 (sequence ID: AF527773) (Fig. 3), which is a very high score given that the data originated from a mixed sequence. From the data it was concluded that both species had been present on the Brucella blood agar and in the blood culture bottles initially. At the time of identification of the blood culture isolates, the urine sample was unfortunately no longer available for further investigation, including anaerobic culturing.

Antimicrobial susceptibility testing with gradient strips (Etest, bioMerieux, Craponne, France and MICE, Oxoid, Basingstoke, UK) was performed on Brucella blood agar for the *F. sanguinis* isolate. It was incubated under anaerobic conditions and read after 24 and 48 h of incubation. The isolate was resistant to metronidazole using European Committee on Antimicrobial Susceptibility Testing (EUCAST) clinical breakpoints for Gram-positive anaerobes (Table 1). It was not possible to reculture the *M. indoligenes* isolate for further testing, but it was concluded that it was metronidazole- and piperacillin/tazobactam-susceptible based on the initial disk diffusion test.

**Table 1.** Antimicrobial susceptibility testing of *F. sanguinis *. Minimum inhibitory concentrations (MICs) were established using gradient strips (Etest and MICE)

Antimicrobial agent	MIC (mg l^−1^)	EUCAST MIC breakpoint S≤/R> (mg l^−1^)
Benzylpenicillin (Etest)	0.006	0.25/0.5
Meropenem (MICE)	0.06	2/8
Moxifloxacin (Etest)	4	na
Vancomycin (Etest)	1	2/2
Clindamycin (MICE)	<0.015	4/4
Tigecycline (Etest)	<0.016	na
Metronidazole (Etest)	>256	4/4

na, Not available

Treatment of the patient was changed to intravenous piperacillin/tazobactam 4 g and metronidazole 500 mg three times a day after the primary finding of *M. indoligenes *. A computed tomography (CT) examination of the abdomen and the urinary tract was performed. Moderate hydronephrosis and hydroureter bilaterally was demonstrated. The patient recovered and was discharged from hospital after 7 days of antibiotic treatment. A flexible cystoscopy was performed after discharge and the prostate was found to be irregular but prostate-specific antigen (PSA) was normal (<0.03 µg l^−1^). As the primary site of infection and cause of bacteraemia had not been established and the patient was diagnosed with an irregular prostate and pain in the back, a positron emission tomography with CT (PET/CT) scan was performed. The PET/CT revealed cancer in the prostate with spread to the bladder and retroperitoneal, infraclavicular and cervical lymph nodes and metastases in the lungs. The cancer was diagnosed as a small-cell neuroendocrine prostate carcinoma.

## Discussion

*M. indoligenes* was described as a new species of a new genus in 2007 by Carlier *et al*. [3]. It is a strict anaerobic bacterium belonging to the family *Lachnospiraceae* in the order *Clostridiales *. The bacterium is a small non-motile Gram-positive rod but can appear Gram-negative. The nearest phylogenetic relatives are *Clostridium* species, which are a part of the human microbial flora in the intestinal tract and species in the family *Lachnospiraceae *, which are found in the human oral cavity [3, 4]. *M. indoligenes* is a non-spore-former, which separate the species from other *Clostridium* species. It has been reported to be susceptible to β-lactam antibiotics and metronidazole. It has previously been isolated from abscesses located intra-abdominally in the buttock and in the thigh [3]. Bacteraemia with *M. indoligenes* has not been described before. *F. sanguinis* has previously been described in a few human cases, including bacteraemia (Beauruelle *et al*. and Falsen *et al*.) [5, 6]. It is a non-spore-forming anaerobic Gram-positive coccus. It has been isolated from deep tissue and joint infections and is believed to be a part of the human surface microbiome. It has previously been reported as sensitive to vancomycin and resistant to metronidazole (as demonstrated with the isolate from this case). *F. sanguinis* is currently not included in the MALDI-TOF database for the Biotyper or VITEK MS systems (VITEK MS, Knowledge base 3.2.0, bioMérieux Nordic).

The species isolated in the presented case of bacteraemia are anaerobic species, which are difficult to culture, isolate and identify, and have only been described in a few clinical cases. It is extremely important to report these kinds of findings with newly described species, so that the next patient with bacteraemia with *M. indoligenes* or *F. sanguinis* can benefit from the observations from the presented case. In a paper by Sabat *et al*. using ‘targeted next-generation sequencing of the 16 S-23S rRNA region for culture-independent bacterial identification’, both *F. sanguinis* and a *Moryella* sp. were detected in urine samples [7]. In this case the primary symptoms and a prostate cancer involving the bladder suggest that the urinary tract could be the primary site of infection.
